# Comparison of 2 PCR assays on environmental samples cultured for *Mycobacterium avium* subsp. *paratuberculosis*

**DOI:** 10.1177/10406387231203970

**Published:** 2023-10-18

**Authors:** Juan Carlos Arango-Sabogal, Olivia Labrecque, Julie-Hélène Fairbrother, Sébastien Buczinski, Jean-Philippe Roy, Julie Arsenault, Vincent Wellemans, Gilles Fecteau

**Affiliations:** Departments of Pathology and Microbiology, Faculty of Veterinary Medicine, Université de Montréal, Saint-Hyacinthe, Québec, Canada; Laboratory of Epidemiological Animal Surveillance of Québec, Ministry of Agriculture, Fisheries and Food of Québec, Saint-Hyacinthe, Québec, Canada; Laboratory of Epidemiological Animal Surveillance of Québec, Ministry of Agriculture, Fisheries and Food of Québec, Saint-Hyacinthe, Québec, Canada; Clinical Sciences, Faculty of Veterinary Medicine, Université de Montréal, Saint-Hyacinthe, Québec, Canada; Clinical Sciences, Faculty of Veterinary Medicine, Université de Montréal, Saint-Hyacinthe, Québec, Canada; Departments of Pathology and Microbiology, Faculty of Veterinary Medicine, Université de Montréal, Saint-Hyacinthe, Québec, Canada; Clinical Sciences, Faculty of Veterinary Medicine, Université de Montréal, Saint-Hyacinthe, Québec, Canada; Clinical Sciences, Faculty of Veterinary Medicine, Université de Montréal, Saint-Hyacinthe, Québec, Canada

**Keywords:** cattle, environmental cultures, IS*900*, ISMap*02*, *Mycobacterium avium* subsp. *paratuberculosis*, PCR

## Abstract

*Mycobacterium avium* subsp. *paratuberculosis* (MAP) is the causal agent of paratuberculosis, a chronic, contagious, and incurable enteric disease of ruminants. An in-house IS*900* PCR assay validated for MAP detection in sheep has been shown to have a higher sensitivity than a commercial PCR and fecal culture. We have now compared the performance of this in-house IS*900* PCR assay with a commercial ISMap*02* PCR assay for the detection of MAP DNA in bovine dairy farm environmental samples. We purposefully selected 30 culture-positive, 62 culture-negative, and 62 non-interpretable environmental samples. We applied the IS*900* PCR assay directly to the frozen inoculum of these samples. Inocula were incubated in an automated system, and growth was confirmed by an acid-fast bacilli stain and the IS*900* PCR assay. Among culture-positive samples before incubation, the IS*900* PCR assay yielded significantly more positive results than the ISMap*02* PCR assay; however, among culture-negative samples, the IS*900* PCR assay yielded positive results both before and after incubation. The ISMap*02* PCR assay did not flag positively among the culture-negative samples either before or after incubation. The IS*900* PCR assay is a sensitive method that can be used to detect MAP DNA in environmental samples before incubation. The ISMap*02* PCR assay is a specific method used to detect MAP DNA in environmental samples both before and after incubation.

Paratuberculosis is a chronic, contagious, and incurable enteric disease of ruminants caused by *Mycobacterium avium* subsp. *paratuberculosis* (MAP). This disease is of worldwide distribution^15^ and is associated with important economic losses to the dairy industry.^12,14,16^ Bacterial culture is the reference antemortem detection test for MAP infections^27^ and can be performed on fecal samples collected from cows (pooled or not) or on environmental samples collected on farms. The major advantage of bacterial culture over other MAP detection tests (e.g., PCR, ELISA) is that a positive result confirms the presence of viable MAP.^6^ However, bacterial culture is more expensive and has a longer turnaround laboratory time than PCR assays and ELISAs. Isolation of MAP is performed using a selective culture medium that can be either solid or liquid. Growth of MAP is faster in liquid media (8–12 wk) than in solid media (10–20 wk).^27^ Even if liquid media are more sensitive than solid media, MAP must be confirmed by PCR because MAP colonies cannot be identified visually in liquid media.^27^

Fecal microbiota may interfere with MAP growth, regardless of the type of sample (individual feces or environmental sample) or the culture medium.^4,20^ Identification of MAP with acid-fast bacilli (AFB) stain is hampered if samples are overgrown by other microorganisms.^27^ As a consequence of microbial overgrowth, samples are declared non-interpretable (NI).^4,20^ A NI result does not provide useful information about the status of the sample, and constitutes a loss of time and money.

Studies have suggested comparable performance of PCR (sensitivity = 60%, specificity = 97%) and culture,^1^ and excellent correlation between quantitative real-time PCR (qPCR) quantification cycle (Cq) and MAP colony-forming units (CFU) in bacterial culture (Spearman rank correlation coefficient = −0.76).^2^ One of the factors that may affect the accuracy of a PCR assay is the choice of the insertion sequence. Among the insertion sequences used for MAP PCR and typing, 3 (IS*900*, f57, ISMap*02*) have been identified to be effective for differentiating MAP from other *M. avium* subspecies.^26^ The IS*900* element is commonly used, and PCR assays based on this element are considered sensitive given the presence of 14–20 copies of the element in the MAP genome.^19,26^ However, it has been suggested that the IS*900* element is not specific to MAP given that it has been found in other environmental mycobacteria.^7,8^ The f57 and ISMap*02* elements are present in 1 and 6 copies in the MAP genome, respectively.^19^

A study that compared the ISMap*02* and the IS*900* elements in conventional and real-time PCR assays for detecting MAP in bovine fecal samples did not find significant differences in the analytical sensitivity and specificity of either element.^24^ However, another study reported the isolation of non-MAP mycobacteria from 2 samples positive by an ISMap*02* PCR assay.^18^ The sequencing of the 16S rRNA and *hsp65* genes suggested that the 2 isolates were likely *M. virginiense* and *M. nonchromogenicum*.^18^ We evaluated the performance of a PCR assay targeting the ISMap*02* element for the identification of MAP DNA on overgrown environmental samples cultured for MAP.^4^ In that study, 62 NI environmental samples were matched by farm, season, and environmental site with 62 MAP culture–negative samples.^4^ In addition, 30 MAP culture–positive environmental samples were analyzed^4^; the PCR assay was applied before and after incubation of samples in the automated system.^4^ The ISMap*02* PCR assay found MAP DNA in 1.6% of the NI samples, and was a specific (among the negative samples) and reliable confirmatory method (among the positive samples).^4^

An in-house PCR assay targeting the IS*900* element was validated for the detection of MAP in culled sheep.^5^ The in-house IS*900* PCR assay had a significantly higher sensitivity (84%) compared to a commercial PCR assay, a commercial ELISA, and fecal culture.^5^ Given that the IS*900* element has also been used for MAP DNA detection in cattle^13,17,23^ and that the environmental sampling strategy has been considered the most cost-effective method to determine MAP herd status,^25^ we compared and report here the performance of the in-house IS*900* PCR and the ISMap*02* PCR assay for the detection of MAP DNA in environmental samples. We hypothesized that both methods have comparable specificity. However, we expected the IS*900* element to have higher sensitivity than the ISMap*02* element for the detection of MAP DNA in environmental samples. Therefore, our objective was to compare the performance of an in-house IS*900* PCR assay and a commercial ISMap*02* PCR assay for the detection of MAP DNA in environmental samples cultured for MAP. Our results may provide evidence that will bridge a research gap regarding the herd sensitivity of PCR testing of environmental samples.^9^

## Materials and methods

### Sample selection and study groups

**Figure 1.**
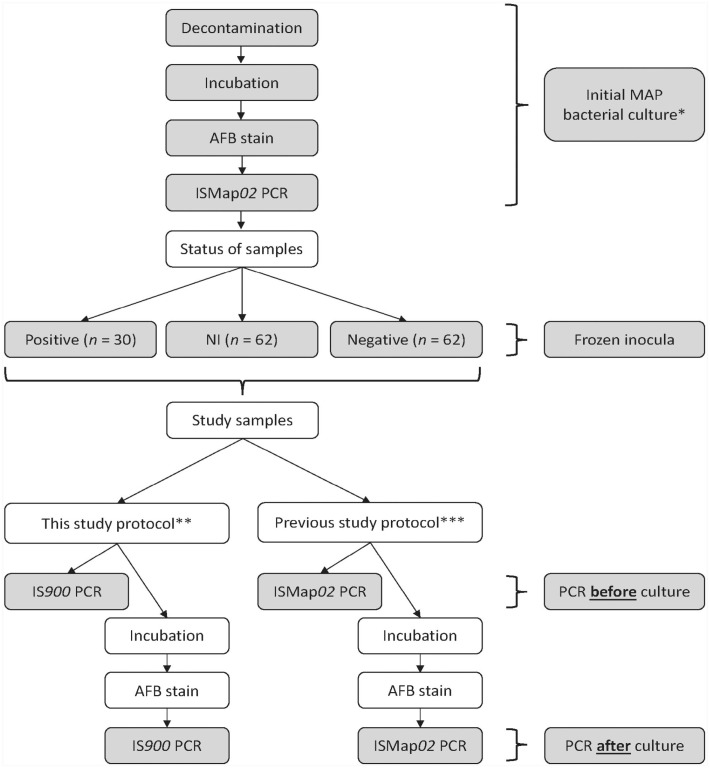
Flowchart of the study protocols to compare 2 PCR methods to detect *Mycobacterium avium* subsp. *paratuberculosis* (MAP) in environmental samples collected from Québec bovine dairy herds when applied before and after incubation in a commercial automated culture system. * The status of the samples analyzed in our study was determined in a 2011 study^3^ by bacterial culture using MGIT Para TB culture medium (BD) and the BACTEC MGIT 960 system (BD). Samples were declared MAP culture–positive if positive by incubation in the automated system, AFB staining (TB fluorescent stain kit; Fisher Scientific), and the ISMap*02* PCR (TaqMan MAP [Johne] reagents; Applied Biosystems). Samples were declared non-interpretable (NI) if positive by incubation in the automated system and negative by AFB stain. Samples were declared negative if no growth was observed in the automated system. ** The IS*900* PCR was applied directly on the frozen inoculum of all samples. Then, all the inocula were incubated in the automated system; growth was confirmed by AFB stain and the IS*900* PCR. IS*900* PCR = in-house system: ABI Fast TaqMan kit (Applied Biosystems); DNA extraction by ZR Fecal DNA extraction system (Zymo Research); target gene: IS*900*. *** The ISMap*02* PCR^4^ was applied directly on the frozen inoculum of all samples. Then, all the inocula were incubated in the automated system; growth was confirmed by AFB stain and the ISMap*02* PCR. ISMap*02* PCR = commercial system: TaqMan MAP (Johne) reagents (Applied Biosystems); DNA extraction by automated nucleic acid isolation (MagMax total nucleic isolation kit; Applied Biosystems); target gene: ISMap*02*.

Environmental samples from our previous study^3^ that aimed to standardize bacterial culture of environmental samples to detect MAP in tie-stall dairy herds were purposefully selected and analyzed as described previously.^4^ Samples were collected in 2011, from different sites of manure accumulation in 23 tie-stall dairy herds distributed in 4 regions of Québec, Canada.^3^ After collection, samples were kept frozen at −70°C until they were cultured for MAP at the Québec Animal Disease Surveillance Laboratory (LEAQ; Saint-Hyacinthe, Québec, Canada). A stratified sampling method was used to select samples according to the bacterial culture results obtained in 2011 to constitute the study groups. We analyzed 62 NI, 62 culture-negative, and 30 culture-positive samples (Fig. 1). NI and negative samples were matched by farm, season, and environmental site.^4^

### Initial MAP culture performed in 2011

The initial isolation of MAP (Fig. 1) was performed using a liquid medium (MGIT Para TB culture medium; BD) and an automated system (BACTEC MGIT 960 system; BD) as reported previously,^3^ following the recommendations of the manufacturer and the U.S. Department of Agriculture (USDA).^11^ As described previously,^3^ following a 3-d decontamination process, 3 tests were carried out in series: 1) incubation in the automated system; 2) AFB staining (TB fluorescent stain kit; Fischer Scientific); and 3) confirmatory ISMap*02* PCR^10^ assay on incubated samples (TaqMan MAP [Johne] reagents, Applied Biosystems; Fig. 1). The AFB stain was performed on samples flagged positive by the automated system. The incubated sample was applied to a slide, heat-fixed at 65–75°C for 2 h, stained with auramine–rhodamine for 15 min, washed with distilled water, destained with alcohol-acid for 10 min, washed with distilled water, and counterstained with potassium permanganate. The slide was then washed, dried, and examined by fluorescence microscopy by a trained technician. For samples positive by AFB staining, DNA was extracted (MagMax total nucleic isolation kit; Applied Biosystems), and a confirmatory ISMap*02* PCR assay was performed (TaqMan MAP [Johne] reagents; Applied Biosystems).

We interpreted the result of these 3 tests according to USDA recommendations: 1) MAP-negative sample = absence of growth; 2) NI sample = positive result by the automated system and negative by AFB stain; 3) mycobacteria other than MAP = positive result by the automated system and by AFB stain but negative by PCR; and 4) MAP-positive sample = positive result by the automated system, AFB stain, and PCR.^4,11,20^

### Study protocol

We performed our study protocol between January and April 2014 in the same laboratory (LEAQ) and at the same time as the study reported in 2016^4^ in which we evaluated the performance of a PCR assay targeting the ISMap*02* element to identify MAP DNA in overgrown environmental samples cultured for MAP (Fig. 1). Briefly, after decontamination in 2011, the inoculum of all samples was kept frozen at −70°C for an average of 802 d (range: 610–971 d) before conducting our 2016 study.^4^ The inoculum of all samples was thawed, and the in-house IS*900* PCR and the ISMap*02* PCRs were applied directly. Then, all the inocula were incubated in the automated system (BACTEC MGIT 960 system; BD); growth was confirmed by AFB staining; the in-house IS*900* PCR assay and the ISMap*02* PCR assay were applied to all the samples regardless of the results of the previous tests (Fig. 1).^4^ A detailed description of the in-house IS*900* PCR assay has been presented previously.^5^ Briefly, this method includes DNA extraction (ZR Fecal DNA extraction system; Zymo Research); an adaptation^13^ of the TaqMan IS*900* PCR assay, primers IS900-for (5′-TGCTGATCGCCTTGCTA-3′) and IS900-rev (5′-GGGCCTGATCGGCGATGAT-3′) as well as probe IS900 (5′-FAM-CCGGGCAGCGGCTGCTTTATATTC-3′-BHQ1), and a PCR reaction (ABI Fast TaqMan kit; Applied Biosystems). The final reaction volume of 25 µL contained 5.5 µL of nuclease-free water, 12.5 µL of master mix, 0.75 µL of primer IS900-for and IS900-rev, 5.5 µL of probe IS900, and 5.0 µL of DNA. The following cycling conditions were used: initial activation at 95°C for 10 min followed by 45 cycles of a 2-step PCR consisting of 95°C for 15 s and 62°C for 60 s. Each PCR run included both positive and negative controls. For both PCR assays (IS*900* and ISMap*02*), a Cq value was used to establish a threshold to discriminate positive (Cq < 37), and suspect (Cq ≥ 37) results, from negative results.^4^

### Statistical analyses

We compared the results obtained by the IS*900* PCR assay and those reported previously for the ISMap*02* PCR assay.^4^ The performance of the 2 PCR assays within each group (i.e., NI, culture-positive, culture-negative) was compared using the McNemar test for paired data. The mean Cq and 95% CI by group were estimated for all of the PCR-positive samples (Cq < 37) before and after the incubation. For all of the samples with available Cq values before and after incubation, the relative change in the amount of MAP DNA after incubation was assessed by converting the Cq values into fold change using the 2^-ΔCq^ method,^22^ in which ΔCq = (Cq after incubation – Cq before incubation). The mean change in MAP DNA by group after incubation was calculated using the following formula:

$$\bar{2^{-\Delta Cq}}=\frac{1}{n}\sum_{i=1}^{n}2^{-\Delta Cq}$$

## Results

Overall, the IS*900* PCR assay yielded more positive results than the ISMap*02* PCR assay (Table 1). Among culture-positive samples (*n* = 30), the IS*900* PCR assay yielded significantly more positive results (*n* = 20; 67%) than the ISMap*02* PCR assay (*n* = 11; 37%) when applied before incubation in the automated system (*p* = 0.007). The mean Cq value (95% CI) for 20 positive samples by the IS*900* PCR assay before incubation was 33.7 (32.6–34.8; min. = 26.8, max. = 37.0); for 11 samples positive by the ISMap*02* PCR assay, it was 35.0 (33.4–36.4; min. = 29.4, max. = 36.9; Suppl. Table 1).

**Table 1.** Results of 2 PCR methods to detect *Mycobacterium avium* subsp. *paratuberculosis* in environmental samples collected from Québec dairy herds when applied before and after incubation in a commercial automated culture system.

Group (*n*)	PCR result before incubation		Culture results		PCR result after incubation		*n*
	IS*900*	ISMap*02*	Incubation	AFB stain	IS*900*	ISMap*02*	
Positive (30)	+	+	+	+	+	+	10
	–	+	+	+	+	+	1
	+	–	+	+	+	+	3
	+	Suspect	+	+	+	+	3
	–	–	+	+	+	+	2
	Suspect	–	+	+	+	+	1
	–	–	+	–	Suspect	–	1
	–	–	+	–	+	–	1
	Suspect	–	+	–	–	–	1
	+	–	+	–	–	–	1
	–	–	+	–	–	–	1
	Suspect	Suspect	+	+	+	+	1
	+	–	–	NA	+	–	1
	+	–	–	NA	–	–	1
	–	–	–	NA	–	–	1
	+	Suspect	–	NA	–	–	1
Negative (62)	–	–	–	NA	–	–	36
	–	–	+	–	–	–	20
	+	–	–	NA	–	–	3
	Suspect	–	–	NA	–	–	2
	Suspect	–	–	NA	+	–	1
NI (62)	–	–	+	–	–	–	33
	–	–	–	NA	–	–	24
	+	+	+	+	+	+	1
	–	–	+	+	–	–	1
	Suspect	–	–	NA	+	–	1
	Suspect	–	+	–	–	–	1
	Suspect	–	–	NA	–	–	1

Automated system = BACTEC MGIT 960 system (BD); AFB = acid-fast bacilli; Group = the known status of the samples as determined by bacterial culture; a quantification cycle (Cq) value was used to establish a threshold to discriminate positive (Cq < 37), suspect (Cq ≥ 37), and negative results; IS*900* PCR = in-house system: ABI Fast TaqMan kit (Applied Biosystems), DNA extraction by ZR Fecal DNA extraction system (Zymo Research), target gene: IS*900*; ISMap*02* PCR = commercial system: TaqMan MAP (Johne) reagents (Applied Biosystems), DNA extraction by automated nucleic acid isolation (MagMax total nucleic isolation kit, Applied Biosystems), target gene: ISMap*02*; NA = not applicable; NI = non-interpretable.

Among the 30 culture-positive samples, after incubation, we observed no statistical difference between the proportion of positive results by the IS*900* PCR assay (*n* = 23; 77%) and the ISMap*02* PCR assay (*n* = 21, 70%; Table 1). The mean Cq value for 23 samples positive by the IS*900* PCR assay after incubation was 29.4 (95% CI: 28.2–30.7; min. = 24.2, max. = 36.6); for the 21 samples positive by the ISMap*02* PCR assay, the mean Cq value was 30.6 (95% CI: 30.0–31.1; min. = 28.0, max. = 33.1; Suppl. Table 1).

Ten culture-positive samples were positive by both PCR assays before and after incubation. The mean Cq value before incubation was 32.4 (95% CI: 30.6–34.3) for the IS*900* PCR assay and 34.9 (95% CI: 33.2–36.5) for the ISMap*02* PCR assay. After incubation, the mean Cq value was 28.2 (95% CI: 26.8–29.7) for the IS*900* PCR assay and 30.1 (95% CI: 29.3–31.0) for the ISMap*02* PCR assay. In 3 culture-positive samples, MAP DNA was not detected by either of the PCR assays either before or after the incubation.

Among culture-negative samples (*n* = 62), the IS*900* PCR assay yielded positive results before (3 samples; 4.8%) and after incubation (1 sample, 1.6%; Table 1). None of these samples was positive by the ISMap*02* PCR assay at any of the steps (before or after) of our study protocol (Table 1). Further, the ISMap*02* PCR assay did not yield positive results either before or after incubation on any of the culture-negative samples (Table 1).

Among the 62 NI samples, sample NI-1 (1.6%) was positive by both PCR assays before and after incubation; sample NI-3 (1.6%) was positive only by the IS*900* PCR assay after incubation. This sample originated from an infected herd in which MAP was not isolated from concomitant individual and environmental samples (Table 2).

**Table 2.** Test results (culture and PCR) and herd characteristics of 10 non-interpretable (NI) and culture-negative environmental samples positive (bold) or suspect to at least one PCR assay applied before and/or after the culture for *Mycobacterium avium* subsp. *paratuberculosis* collected from Québec dairy herds.

Group (according to 2011 results)-sample ID	Test results in 2014									
	PCR* before incubation (Cq)				PCR* after incubation (Cq)		Herd characteristics in 2011†			
	IS*900*	ISMap*02*	Incubation	AFB stain	IS*900*	ISMap*02*	Herd size	Fecal WHP, %	No. of positive samples/No. of samples	Historical herd status
NI-1	**34.3**	**36.0**	+	+	**28.3**	**30.5**	35	28.6	7/10	+
NI-2	39.0	–	+	–	–	–	72	5.6	1/9	+
NI-3	39.8	–	–	NA	**34.5**	–	62	0	0/9	+
NI-4	37.7	–	–	NA	–	–	179	0.6	0/9	+
Negative-1	**27.6**	–	–	NA	–	–	46	0	0/9	+
Negative-2	38.0	–	–	NA	–	–	179	0.6	0/9	+
Negative-3	**36.6**	–	–	NA	–	–	64	0	0/9	+
Negative-4	37.9	–	–	NA	–	–	63	0	0/9	–
Negative-5	**35.2**	–	–	NA	–	–	56	3.6	6/9	+
Negative-6	39.9	–	–	NA	**36.2**	–	47	0	0/9	+

Automated system = BACTEC MGIT 960 system (BD); AFB = acid-fast bacilli; NA = not applicable. Bold characters indicate PCR positive samples (Cq <37).

* IS*900* PCR = in-house system: ABI Fast TaqMan kit (Applied Biosystems); DNA extraction by ZR Fecal DNA extraction system (Zymo Research); target gene: IS*900*. ISMap*02* PCR = commercial system: TaqMan MAP (Johne) reagents (Applied Biosystems); DNA extraction by automated nucleic acid isolation (MagMax total nucleic isolation kit, Applied Biosystems); target gene: ISMap*02*.

† According to Arango-Sabogal et al.^4^ WHP = within-herd prevalence based on individual fecal culture. Historical herd status was based on individual or environmental culture results (positive if *Mycobacterium avium* subsp. *paratuberculosis* was cultured from at least 1 sample within 24 mo before the study or during the study period; negative if it had 2 negative culture results of environmental samples [sampled in a 12–18-mo interval] and no clinical animals [persistent diarrhea and loss of body weight and normal appetite] during the 24 mo before the study).

Samples with available Cq values before and after incubation were analyzed to calculate the increase of MAP DNA after incubation. Among the culture-positive samples, the average DNA increase observed using the IS*900* PCR assay was 149-fold (*n* = 19; 95% CI: 15.4–283), and using the ISMap*02* PCR assay was 66-fold (*n* = 15; 95% CI: 27.7–106.1). For the only culture-negative sample found positive by the IS*900* PCR assay before and after incubation, a 26-fold increase in DNA was observed. The MAP DNA increase was similar for the NI sample found positive by both PCR assays (IS*900* PCR = 59-fold; ISMap*02* PCR = 58-fold). For the remaining NI sample positive by the IS*900* PCR assay before and after incubation, we observed a 38-fold MAP DNA increase (Suppl. Table 1).

## Discussion

A key finding of our study was the higher sensitivity of the IS*900* PCR assay compared to the ISMap*02* PCR assay for the detection of MAP DNA in culture-positive environmental samples before the incubation of samples in the automated system. This finding is supported by the significant difference in the number of culture-positive samples that were confirmed by the IS*900* PCR assay compared with the ISMap*02* PCR assay. Our other study findings also suggest higher analytical sensitivity of the IS*900* PCR assay compared to the ISMap*02* PCR assay. Specifically, the Cq values of the IS*900* PCR assay tended to be lower than those obtained by the ISMap*02* PCR assay, and a higher mean DNA increase was observed for the IS*900* PCR assay compared to the ISMap*02* PCR assay among samples with all Cq values available.

One of the factors that may impact the analytical sensitivity of PCR assays is the insertion sequence targeted by the assay.^19^ It has been suggested that the IS*900* insertion sequence can be used to increase the sensitivity of the PCR assays^26^ given that it is present in 14–20 copies in the MAP genome.^19^ Our observation of higher sensitivity of the IS*900* PCR assay relative to the ISMap*02* PCR assay before incubation suggests that the IS*900* PCR assay may be a useful method to detect MAP DNA directly in environmental samples. This option may contribute to a faster laboratory turnaround time compared to bacterial culture. However, care must be taken when extrapolating our results because the substrate matrix that we used was the decontaminated inoculum resulting from the 3-d decontamination process and not fresh environmental samples.

Our results suggest lower specificity of the IS*900* PCR assay compared to the ISMap*02* PCR assay. Four culture-negative samples were positive by the IS*900* PCR assay; none of the culture-negative samples were found positive by the ISMap*02* PCR assay. Using a Bayesian latent class model, median diagnostic specificity of an IS*900* PCR assay to detect MAP DNA in bovine fecal samples has been estimated to be ~96% for prevalence scenarios of 20–70%.^21^ The high specificity of ISMap*02* that we observed might be explained by the specificity of the target gene. In a previous study, PCR methods using ISMap*02* did not amplify DNA of any of the genetically close mycobacteria.^24^ Although one study questioned the specificity of the PCR methods using the ISMap*02* assay,^18^ it seems very unlikely that viable MAP would be present in the 4 culture-negative samples that were positive by the IS*900* PCR assay. Both incubations (initial incubation performed in 2011, and incubation for the present study protocol) were negative. The results analysis of concomitant herd tests and herd characteristics^3^ (Table 2) of the 4 culture-negative samples (samples Negative-1, Negative-3, Negative-5, Negative-6; Table 2) positive by the IS*900* PCR assay (before or after incubation) suggests that, at least in 3 of the samples (Negative-1, Negative-3, Negative-6; Table 2), MAP DNA was not present. Although these samples (Negative-1, Negative-3, Negative-6; Table 2) originated from herds with a positive historical status,^3^ MAP was not isolated from other concomitant individual and environmental samples collected from these herds at the same sampling visit^3^ (Table 2).

Concomitant positive individual (MAP within-herd prevalence = 3.6%) and environmental (6 of 9 positive) samples were observed in the herd from which the remaining culture-negative sample (Negative-5; Table 2) positive by the IS*900* PCR assay was obtained.^3^ If MAP DNA was indeed present in this sample, this result indicates a lack of sensitivity (false-negative result) of the ISMap*02* PCR assay. This result could have also been a consequence of the higher sensitivity of the IS*900* PCR assay compared to the ISMap*02* PCR assay if the viability of MAP was affected by the 3-d decontamination protocol of the culture performed in 2011. Another explanation for this discordant result may be the detection of environmental mycobacteria by the IS*900* PCR assay (i.e., a lack of specificity of the IS*900* PCR assay). It has been suggested that false-positive results can be obtained with PCR methods, including the IS*900*, because this insertion sequence is present in other environmental mycobacteria and is not unique to MAP.^8^

Among the NI samples, both PCR assays agreed on the positive results for one sample before and after incubation. According to the herd test results and herd characteristics (Table 2), this sample is likely a MAP-positive sample. However, discordant results between the PCR methods were also observed among NI samples (i.e., one NI sample was positive only by the IS*900* PCR assay after incubation). Given that no concomitant positive cultures were observed in the farm from which this sample was collected, this could be a false-positive result of the IS*900* PCR assay. One study comparing the use of the insertion sequences IS*900* and ISMap*02* for detecting MAP DNA in bovine fecal samples observed a higher specificity of PCR assays using ISMap*02* and comparable sensitivity to the 2 methods.^24^ Most of our results agree with this study. The specificity of the ISMap*02* PCR assay was likely higher than that of the IS*900* PCR assay, and the sensitivity of both assays was comparable only after incubation of samples in the automated system. The higher sensitivity of the IS*900* PCR before incubation in the automated system contradicts the results of that previous study.^24^

Further to our results, 2 possible testing strategy scenarios might be considered to take advantage of the benefits of the PCR assays that we used. These scenarios depend on whether bacterial culture is included in the testing process (i.e., if PCR would be used as a confirmatory method) and if the tests are interpreted in series. If bacterial culture is performed, the strategy may include screening with the IS*900* PCR directly on fecal samples, then culturing only the IS*900* PCR–positive samples, and performing the ISMap*02* PCR assay after culture to confirm the status of these samples. In this scenario, a MAP-positive sample would be a sample positive to both the initial (IS*900*) and confirmatory (ISMap*02*) PCR assays regardless of the culture results. This strategy allows an in-crease in the specificity of the entire process. However, the inclusion of the initial screening with the IS*900* PCR assay would involve additional cost compared to bacterial culture. Nevertheless, this initial step may detect samples that might go undetected during the conventional culture process. On the other hand, if bacterial culture is not performed, initial screening of samples using the IS*900* PCR assay and then confirmation of IS*900* PCR–positive samples with the ISMap*02* PCR assay is a possibility. The main advantage of this option is the reductions in both turnaround time and costs. Also, one might expect a gain in specificity of the process. However, the positive impact of incubation on MAP growth would not be present. As we demonstrated, incubation caused a significant increase in MAP DNA that translated into an increase in MAP detection by PCR, which was more evident when using the ISMap*02* PCR assay. The culture-free strategy would not confirm the presence of viable MAP but simply confirm the presence of MAP DNA. However, from a herd control perspective, detecting MAP DNA on the farm is enough evidence to implement prevention and control strategies. The 2 testing strategies presented here are not exhaustive and could be used both in a control program and a research context.

A limitation of our study is the use of frozen decontamination inoculum prepared from environmental samples instead of fresh environmental or fecal samples. We performed our project in collaboration with the LEAQ, a provincial diagnostic laboratory. Given that storage capacity can be limited, we used the decontamination inoculum that had been kept frozen after the culture was performed in 2011. Using this matrix might limit the extrapolation of our results to other matrix types (e.g., fresh environmental samples). Other than that, the samples that we used originated from typical commercial dairy herds, were collected using a standardized environmental sampling strategy,^3^ and were decontaminated and cultured using a standardized protocol.^3,20^

The absence of an observed difference between the proportion of positive results by the IS*900* and ISMap*02* PCR assays among culture-positive samples after incubation should not be interpreted as a similar performance of both PCR methods when applied after culture. The samples that we analyzed were convenience-selected among the available samples from a previous study,^3^ and a sample size and study power calculation were not performed a priori. In the case of culture-positive samples, we included all of the samples that were available.

## Supplemental Material

sj-pdf-1-vdi-10.1177_10406387231203970 – Supplemental material for Comparison of 2 PCR assays on environmental samples cultured for Mycobacterium avium subsp. paratuberculosis Click here for additional data file. Supplemental material, sj-pdf-1-vdi-10.1177_10406387231203970 for Comparison of 2 PCR assays on environmental samples cultured for Mycobacterium avium subsp. paratuberculosis by Juan Carlos Arango-Sabogal, Olivia Labrecque, Julie-Hélène Fairbrother, Sébastien Buczinski, Jean-Philippe Roy, Julie Arsenault, Vincent Wellemans and Gilles Fecteau in Journal of Veterinary Diagnostic Investigation
