# Supplementary material for: Comparison of 2 PCR assays on environmental samples cultured for Mycobacterium avium subsp. paratuberculosis
Source: J Vet Diagn Invest. 2023 Oct 18;36(1):24–31. doi: 10.1177/10406387231203970 (PMC10734583; doi:10.1177/10406387231203970)
Supplement: sj-pdf-1-vdi-10.1177_10406387231203970 – Supplemental material for Comparison of 2 PCR assays on environmental samples cultured for Mycobacterium avium subsp. paratuberculosis [file sj-pdf-1-vdi-10.1177_10406387231203970.pdf]

Arango-Sabogal JC, et al. Comparison of 2 PCR assays on environmental samples cultured for *Mycobacterium avium* subsp. *paratuberculosis*

Supplementary Table 1. Comparative quantification cycle (Cq) values of 2 PCR methods when applied before and after the culture of *Mycobacterium avium* subsp. *paratuberculosis* (MAP) from environmental samples collected from Québec bovine dairy herds.

	PCR results before incubation, Cq		Culture results		PCR results after incubation, Cq		MAP DNA fold increase after incubation, $2^{-\Delta Cq}$	
Group	IS*900*	ISMap*02*	Incubation	AFB stain	IS*900*	ISMap*02*	IS*900*	ISMap*02*
Positive	**35.8**	**36.9**	+	+	**25.8**	**29.9**	1,020	124
	**26.8**	**29.4**	+	+	**24.2**	**28.0**	6.0	2.5
	**32.9**	**36.0**	+	+	**29.4**	**30.6**	11.6	42.5
	**34.6**	**34.5**	+	+	**26.7**	**30.0**	247	22.3
	**33.7**	**35.9**	+	+	**28.7**	**29.5**	31.8	87.4
	**33.5**	**35.4**	+	+	**30.3**	**31.8**	9.1	12.4
	**33.2**	**36.8**	+	+	**29.3**	**31.3**	14.8	45.6
	**32.7**	**36.9**	+	+	**30.7**	**30.9**	4.1	66.7
	**30.8**	**33.4**	+	+	**28.4**	**30.6**	5.3	6.8
	**30.6**	**33.5**	+	+	**29.0**	**28.8**	2.9	26.0
	**33.1**	37.4	+	+	**28.6**	**29.9**	22.9	176

	**35.3**	37.1	+	+	**30.0**	**31.4**	38.3	52.0
	37.4	37.9	+	+	**31.0**	**33.1**	79.3	29.0
	**35.4**	39.9	+	+	**31.9**	**31.8**	11.6	260
	–	**35.8**	+	+	**27.5**	**30.1**	NA	50.6
	37.4	–	+	+	**28.4**	**30.3**	538	NA
	**35.7**	–	+	+	**28.4**	**30.8**	168	NA
	**37.0**	–	+	+	**27.7**	**30.6**	4.4	NA
	**33.9**	–	+	+	**31.7**	**32.5**	0.3	NA
	**34.9**	–	–	NA	**36.5**	–	0.3	NA
	**36.1**	39.1	–	NA	–	–	NA	NA
	–	–	+	+	**28.3**	**29.8**	NA	NA
	–	–	+	+	**28.4**	**30.6**	NA	NA
	–	–	+	–	37.2	–	NA	NA
	–	–	+	–	**36.6**	–	NA	NA
	37.0	–	+	–	–	–	NA	NA
	**35.3**	–	+	–	–	–	NA	NA
	**33.2**	–	–	NA	–	–	NA	NA
Negative	**27.6**	–	–	NA	–	–	NA	NA
	38.0	–	–	NA	–	–	NA	NA
	**36.6**	–	–	NA	–	–	NA	NA
	37.9	–	–	NA	–	–	NA	NA
	**35.2**	–	–	NA	–	–	NA	NA
	39.9	–	–	NA	**36.2**	–	12.8	NA
NI	**34.3**	**36.0**	+	+	**28.3**	**30.5**	59.3	58.1
	39.0	–	+	–	–	–	NA	NA

39.8	–	–	NA	**34.5**	–	38.1	NA
37.7	–	–	NA	–	–	NA	NA

Automated system = BACTEC MGIT 960 system (BD); AFB = acid-fast bacilli; ΔCq = (Cq value after incubation – Cq value before incubation); IS*900* PCR = in-house system: ABI Fast TaqMan kit (Applied Biosystems), DNA extraction by ZR Fecal DNA extraction system (Zymo Research), target gene: IS*900*; ISMap*02* PCR = commercial system: TaqMan MAP (Johne) reagents (Applied Biosystems), DNA extraction by automated nucleic acid isolation (MagMax total nucleic isolation kit, Applied Biosystems), target gene: ISMap*02*; NA = not applicable; NI = non-interpretable. Cq values in bold are positive; non-bold values are suspect. Bold characters indicate PCR-positive samples (Cq <37).
